# Metabolic engineering of *Kluyveromyces lactis* for L-ascorbic acid (vitamin C) biosynthesis

**DOI:** 10.1186/1475-2859-12-59

**Published:** 2013-06-22

**Authors:** Júlio César Câmara Rosa, Lívia Tavares Colombo, Mariana Caroline Tocantins Alvim, Nelson Avonce, Patrick Van Dijck, Flávia Maria Lopes Passos

**Affiliations:** 1 Laboratório de Fisiologia de Microrganismos, Instituto de Biotecnologia Aplicada à Agropecuária (BIOAGRO), Universidade Federal de Viçosa, Brazil; 2 Departamento de Microbiologia, Universidade Federal de Viçosa, campus Viçosa, Minas Gerais, Brasil; 3 Laboratory of Molecular Cell Biology, Institute of Botany and Microbiology, KU, Leuven; 4 Department of Molecular Microbiology, VIB, Kasteelpark Arenberg 31, B-3001 Leuven-Heverlee, Flanders, Belgium; 5 Av. P. H. Rolfs s/nº, 36571-000, Laboratório de Fisiologia de Microrganismos, BIOAGRO, Universidade Federal de Viçosa, Viçosa–MG, Brazil

**Keywords:** *Kluyveromyces lactis*, L-ascorbic acid, L-galactose, Metabolic engineering

## Abstract

**Background:**

L-ascorbic acid (L-AA) is naturally synthesized in plants from D-glucose by 10 steps pathway. The pathway branch to synthesize L-galactose, the key intermediate for L-ascorbic acid biosynthesis, has been recently elucidated. Budding yeast produces an 5-carbon ascorbic acid analogue Dehydro-D-arabinono 1,4-lactone (D-DAL), which is synthesized from D-arabinose. Yeast is able to synthesize L-ascorbic acid only if it is cultivated in the presence of one of its precursors: L-galactose, L-galactono 1,4-lactone, or L-gulono 1,4-lactone extracted from plants or animals. To avoid feeding the yeast culture with this “L” enantiomer, we engineered *Kluyveromyces lactis* with L-galactose biosynthesis pathway genes: GDP-mannose 3,5-epimerase (GME), GDP-L-galactose phosphorylase (VTC2) and L-galactose-1-phosphate phosphatase (VTC4) isolated from *Arabidopsis thaliana*.

**Results:**

Plasmids were constructed and modified such that the cloned plant genes were targeted to the *K. lactis* LAC4 Locus by homologous recombination and that the expression was associated to the growth on D-galactose or lactose. Upon *K. lactis* transformation, GME was under the control of the native LAC4 promoter whereas VTC2 and VTC4 were expressed from the *S. cerevisiae* promoters *GPD1* and *ADH1* respectively. The expression in *K. lactis,* of the L-galactose biosynthesis genes was determined by Reverse Transcriptase-PCR and western blotting. The recombinant yeasts were capable to produce about 30 mg.L^-1^ of L-ascorbic acid in 48 hours of cultivation when cultured on rich medium with 2% (w/v) D-galactose. We also evaluated the L-AA production culturing recombinant recombinant strains in cheese whey, a waste product during cheese production, as an alternative source of lactose.

**Conclusions:**

This work is the first attempt to engineer *K. lactis* cells for L-ascorbic acid biosynthesis by a fermentation process without any trace of “L” isomers precursors in the culture medium. We have engineered *K. lactis* strains capable of converting lactose and D-galactose into L-galactose, by the integration of the genes from the *A. thaliana* L-galactose pathway. L-galactose is a rare sugar, which is one of the main precursors for L-AA production.

## Background

The enediol ascorbate or L-ascorbic acid (L-AA), known as Vitamin C, is an important metabolite in many organisms. In eukaryotes, L-AA is essential for a variety of cellular functions [1], acting as I) a scavenger of free radicals [2]; ii) a reducing agent [3], iii) a cofactor for enzyme activity [4,5] iv) an intermediate for catecholamines biosynthesis, and v) a limiting growth factor in plant development [6]. Most of the commercially available vitamin C is synthetically synthesized by the Reichstein process, using D-glucose as start material [7].

L-AA is naturally produced in plants where its biosynthetic pathway has been completely elucidated [8,9]. In most cases, GDP-D-mannose is converted into L-galactose, which is further converted into L-AA [10]. Although there may exist alternative routes [11,12] this pathway is recognized as the main route for L-AA biosynthesis [13,14]. There are three enzymes required for the conversion of GDP-D-mannose into L-galactose. The GDP-mannose 3,5-epimerase (GME) catalyzes the conversion of GDP-D-mannose to GDP-L-gulose or to GDP-L-galactose, depending whether the epimerization occurs on 5’- carbon or on both 3’- and 5’- carbon of GDP-D-mannose respectively [15]. GDP-L-gulose seems to represent the minor part of the products (around 25% under equilibrium) and can also be converted to L-AA [16]. The epimerization of D to L-substrates, which is rare in nature, is a crucial step to generate the galactose enantiomer in the L-AA pathway. GDP-L-galactose is then converted to L-galactose 1-phosphate by GDP-L-galactose phosphorylase, encoded by the VTC2 gene [17]. This gene encodes a member of the GalT/Apa1 branch of the histidine triad protein superfamily that catalyzes the conversion of GDP-L-galactose to L-galactose 1-phosphate in a reaction that consumes inorganic phosphate and produces GDP [9]. Müller-Moulé [18] constructed the VTC2:YFP fusion protein and unexpectedly this protein was found not only in the cytosol, but also in the nucleus, which suggests that GDP-L-Galactose phosphorylase/L-Galactose guanylyltransferase might be a dual-function protein, which has both enzymatic and regulatory function in the L-AA biosynthesis pathway in *A. thaliana*. The third enzyme is L-galactose 1-phosphate phosphatase, encoded by the VTC4 gene [19], which is a bifunctional enzyme that plays a role in both ascorbate as well as myoinositol biosynthetic pathways, although it shows selective preference for L-galactose 1-phosphate [20]. The resulting L-galactose is then the main precursor for L-AA biosynthesis.

Yeasts are known to produce the 5-carbon ascorbic acid analogue, Dehydro-D-arabinono 1,4-lactone (D-DAL), which is synthesized from D-arabinose. Although D-DAL does not show any anti-scurvy activity, its physiochemical properties and biological activities are quite similar to those of L-AA. For this reason D-DAL can replace L-AA in some industrial applications [21,22]. The structural motifs of the enzymes involved in the D-DAL biosynthetic pathway in yeast resemble those of the pathway in plants that converts L-galactose into L-AA. D-DAL pathway enzymes from *Candida albicans* and *Saccharomyces cerevisiae* have shown to be able to convert a broad range of substrates besides D-arabinose including L-galactose into their respective galactonic acids in vitro [23,24]. Furthermore, L-AA production in yeasts was achieved when appropriate precursors such as L-galactose, L-galactono 1,4-Lactone, L-gulono 1,4-lactone were exogenously supplied in the growth medium [25]. Thus, isolation of genes involved in L-galactose production in plants provides biochemical support to guide the metabolic capacity of industrial microorganisms to produce L-AA by fermentation [7].

Attempts have been made to synthesize L-AA in genetically modified microorganisms. Sauer et al. [25] observed a high production of vitamin C in the culture supernatant of *S. cerevisiae* cells expressing the L-galactose dehydrogenase (LDGH) and D-arabinose 1,4-lactone oxidase (ALO1) from yeast or the L-galactono-1, 4-lactone dehydrogenase (AGD) from *Arabidopsis thaliana,* when cultivated in a medium containing 250 mg.L^-1^ L-galactose. Further, Branduardi et al. [26] have engineered this strain with GME and VTC4 from *A. thaliana* and also with L-fucose guanylyltransferase from *Rattus norvegicus* FGT in order to convert D-glucose to L-AA completing the L-AA pathway in *S. cerevisiae*. The L-AA production conferred an increased stress tolerance under oxidative stress conditions.

*Kluyveromyces lactis* is one of the most important non-*Saccharomyces* yeast species used as an eukaryotic model and tool for biotechnological applications including an alternative host for heterologous gene expression. *K. lactis* has the ability of growing, by respiration, on a wide range of substrates, including lactose with low glucose repression [27]. The genome has been completely sequenced and the Lac-Gal regulon, with the induced genes for lactose transport and hydrolysis, has been extensively studied [28]. Many heterologous expression systems have been developed, based on the LAC4 promoter with the production of lysozyme [29], serum albumin [30], thermostable bacterial xylanase [31] and heparin sulfate sulphotransferase [32] as examples. The potential use of *K. lactis* as a host for protein expression associated to its physiological properties suggests that this yeast could also be used for large-scale protein production in the food and pharmaceutical industry. Furthermore, its ability to express and process heterologous proteins makes this yeast well suited for multiple proteins expression such as the enzymes involved in L-galactose metabolism from plants.

Considering the high costs of using non-physiological substrates in the L enantiomer form for industrial applications, herein, we report the construction of *K. lactis* strains capable to convert D-galactose or lactose into L-galactose, the main intermediate metabolite of the L-AA pathway in plants, and its subsequent conversion into L-ascorbic acid.

## Results

### Isolation and cloning of the L-ascorbic acid pathway genes from *Arabidopsis thaliana*

**Figure 1:**
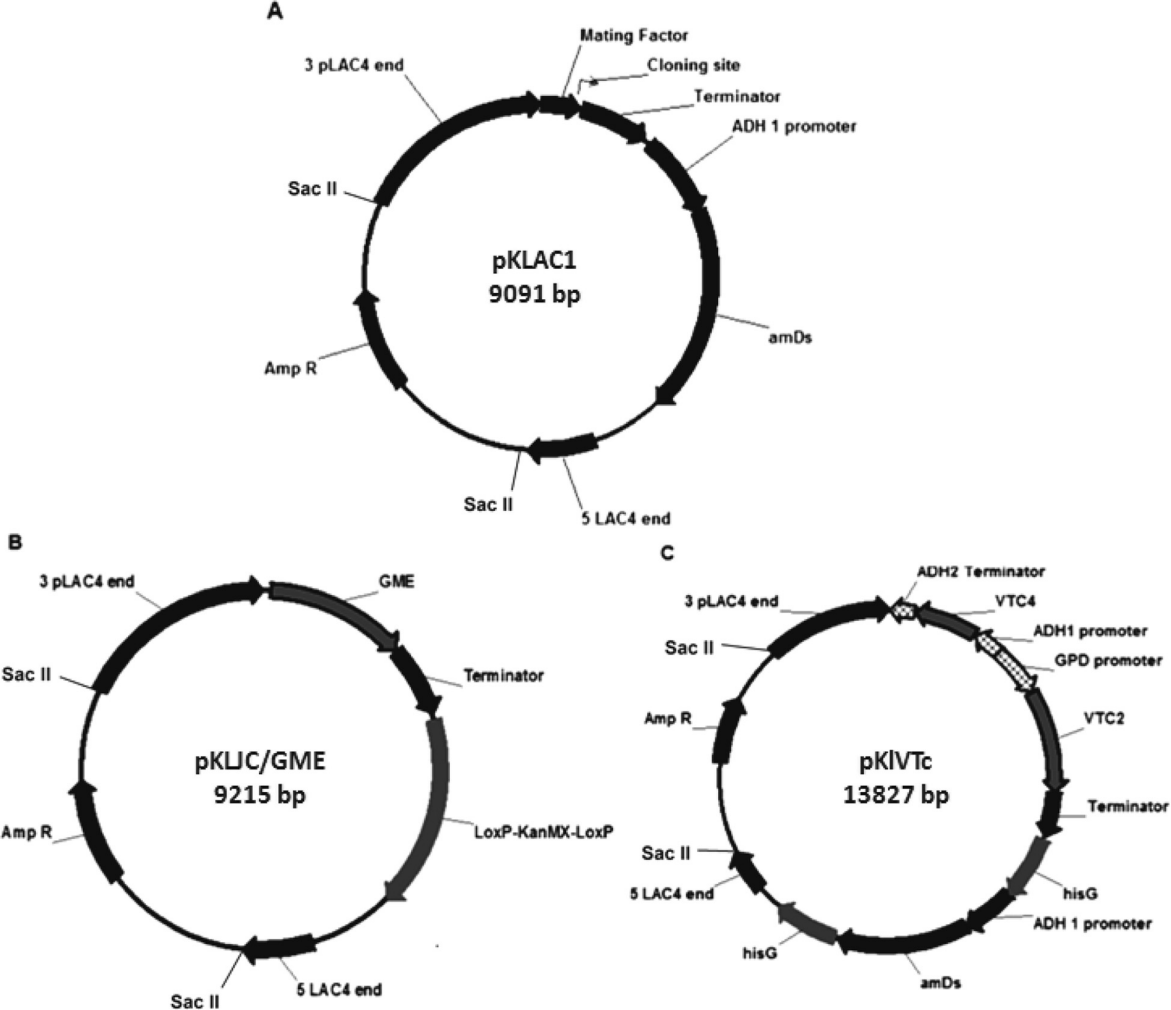
**Maps of the plasmid vectors used for L-AA genes expression.** pKlJC/GME (**B**) and pKlVTc (**C**) vectors are derived from pKLAC1 (**A**). The vectors contain the 5´ and 3´ ends of the LAC4 promoter separated by DNA encoding β-lactamase (Amp^R^) and the pMB1 origin (ori). Plasmid pKlVTc contains a construct where the yeast *ADH1* promoter drives expression of an acetamidase selectable marker gene (*amdS*), which is flanked by hisG direct repeats. *ADH1* and *GPD1* promoters from *Saccharomyces cerevesiae* drive the transcription of AtVTC4 and AtVTC2 respectively. Plasmid *pKLJC*/GME contains the LoxP-KanMX-LoxP cassette that confers resistance to geneticin.

A cDNA library from *Arabidopsis thaliana* leaves was used as template to amplify the three genes of the L-ascorbic acid (L-AA) pathway required for L-galactose synthesis in *K. lactis* (see Materials and Methods). The amino acid sequences encoded by the corresponding amplified genes GME, VTC2, VTC4 were determined and verified to be the same as those in the Arabidopsis genome database. The three genes were cloned in *K. lactis* expression vectors (Figure 1). The codons of the plant genes were not optimized for expressing in *K. lactis*. Carbone et al. (2003) [33] reported that *Saccharomyces* sp. and plants shared the same preferred codons, supporting *K. lactis* as a host for unmodified plant genes expression.

### *Kluyveromyces lactis* strains expressing L-AA genes

**Figure 2:**
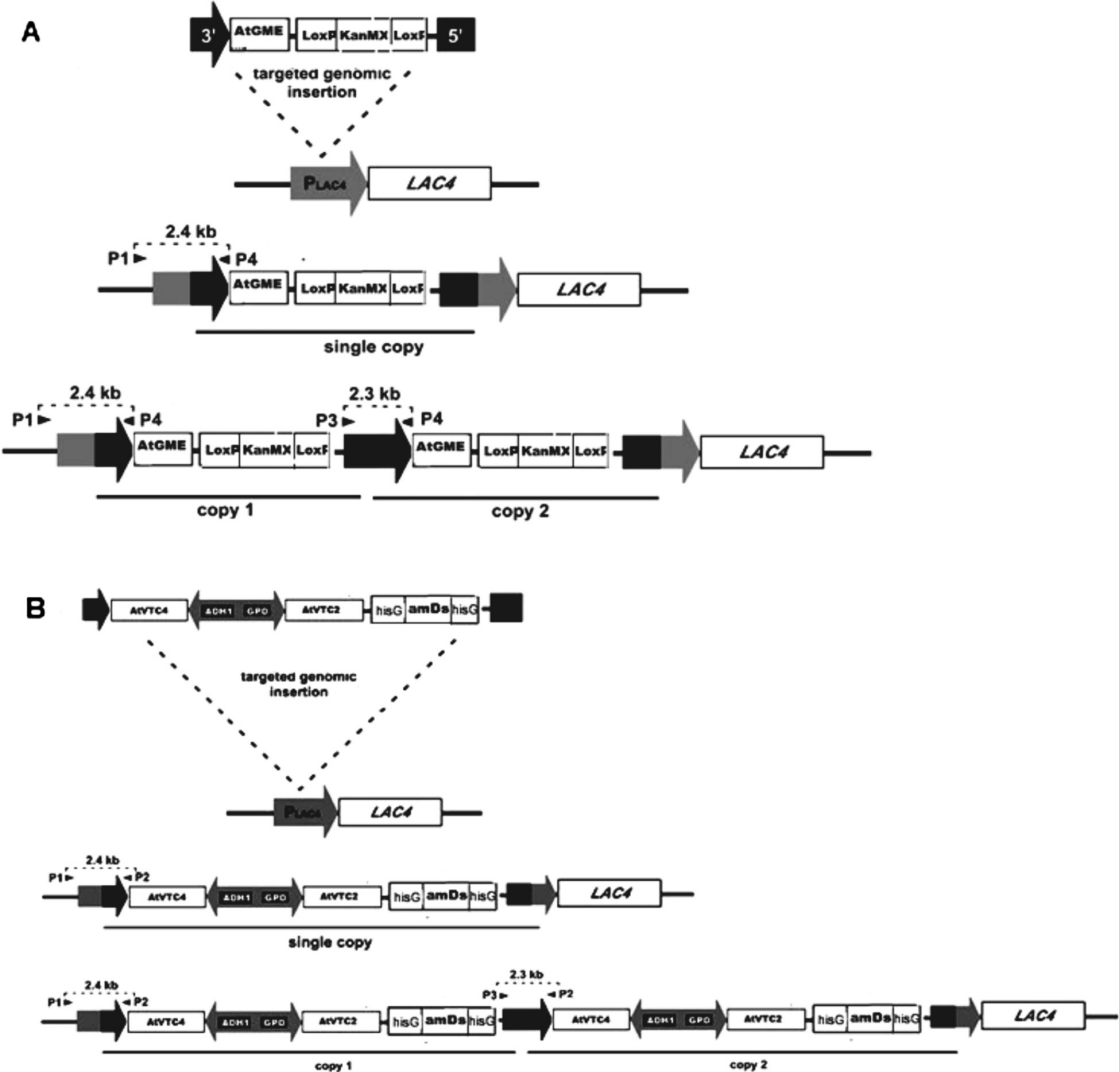
**Genomic organization of SacII linearized vectors into the *K. lactis *LAC4 locus upon transformation.** (**A**) Scheme of single and multiple copies of integrated GME cassettes detected by PCR using primers P1 and P3 (2.4 kb) or P3 and P4 (2.3 kb) respectively; (**B**) Scheme of single and multiple copies of AtVTC2 and AtVTC4 cassettes detected by PCR using primers P1 and P2 (2.4 kb) or P3 and P2 (2.3 kb) respectively.

To obtain strains producing L-AA, *K. lactis* CBS2359 cells were transformed with Sac II linearized pKLJC/GME and pKlVTc vectors constructed in this work (Figure 1). Strain JVC1-5 was obtained by transformation of *K. lactis* CBS2359 cells with the pKLJC/GME vector. The strains JVC1-51, JVC1-53, and JVC1-56 were derived from JVC1-5 by transformation with pKlVtc vector containing the VTC4 and VTC2 expression cassette. The JVC3-18 strain was generated through single-step transformation of *K. lactis* CBS2359 with both plasmids. The JVC2-1 and JVC2-2 strains were constructed by transformation of the *K. lactis* CBS2359 cells with the pKlVTc vector. All yeast strains used in this work are listed in Table 1. The selection of *K. lactis* cells transformed with pKlVtc was achieved by growth on YCB agar medium containing 5 mM acetamide. The vector harbors the *amDs* marker which has been reported to favor transformants with more than one integration event into the genome [34]. Correct integration into the *K. lactis* LAC4 locus was confirmed by PCR analysis using the primers P1, P2, P3 and P4 (Table 2). Figure 2 provides a schematic overview of the resulting genomic organization of the integrated plasmids at the *K. lactis* LAC4 chromosomal locus. Primer P1 was designed to anneal at the chromosomal LAC4 promoter upstream of the vector integration site and the reverse primers P2 and P4 anneal to pKlVTc and pKLJC/GME expression cassettes sequence respectively. When multiple copies of the cassette were integrated in tandem at the same locus, a 2.3 kb fragment was then amplified by using the forward primer P3 in combination with either reverse primers P2 or P4 for each vector. Single and multiple insertions from each cassette were detected by the presence of 2.4 kb and 2.3 kb amplicons respectively. The insertion of the cassette into the LAC4 locus by homologous recombination duplicates the LAC4 promoter region so that it can be targeted by another cassette resulting in multiple copies integration. However, this analysis does not indicate the number of integrated copies; we determined the exact copy number of each cassette integrated into *K. lactis* genome by absolute quantification. The results are shown in Table 3. Most of the recombinant strains harbor at least more than one copy of the cassette except the strain JVC1-51. The JVC1-56 strain carries four copies of the GME gene integrated in tandem at the LAC4 locus.

**Table 1:** Yeasts strains used in this study

**Strains**	**Markers**	**Cassette expression**	**Plasmids**	**Reference**
**CBS 2359**	Wild type	-	-	Genolevures consortium^*^
**JVC1-5**	Kan^R^	AtGME	pKLJC/GME	This study
**JVC1-51**	Kan^R^, amDs	AtGME, AtVTC2, AtVTC4	pKLJC/GME, pKlVTc	This study
**JVC1-53**	Kan^R^, amDs	AtGME, AtVTC2, AtVTC4	pKLJC/GME, pKlVTc	This study
**JVC1-56**	Kan^R^, amDs	AtGME, AtVTC2, AtVTC4	pKLJC/GME, pKlVTc	This study
**JVC2-1**	amDs	AtVTC2, AtVTC4	pKlVTc	This study
**JVC2-2**	amDs	AtVTC2, AtVTC4	pKlVTc	This study
**JVC3-18**	Kan^R^, amDs	AtGME, AtVTC2, AtVTC4	pKLJC/GME, pKlVTc	This study

Kan^R^ cassette conferring resistance to Geneticin.

amDs acetamidase marker.

*Kluyveromyces lactis strain used for Genome sequencing by the Génolures consortium (http://www.genolevures.org).

**Table 2:** List of primers used on this study

**Name**	**Sequence**	**Restriction site**
**GME-F**	5’**CTCGAG**ATGGGAACTACCAATGGAACAG3’	XhoI
**GME-RFlag**	5’**CCCGGCGGCCGTCA**CTTGTCATCGTCATCCTTGTAATCCTCTTTTCCATCAGCCGCG3’	NotI
**VTC2-F**	5’**GCGGCCGC**ATGTTGAAAATCAAAAGAGTTCCGACC3’	NotI
**VTC2-RFlag**	5’**AGGCCTTCA**CTTGTCATCGTCATCCTTGTAATCCTGAAGGACAAGGCACTCGGCGGC3’	StuI
**VTC4-F**	5’**CTCGAG**ATGGCGGACAATGATTCTCTAG3’	XhoI
**VTC4-RFlag**	5’**AGGCCTTCA**CTTGTCATCGTCATCCTTGTAATCTGCCCCTGTAAGCCGC3’	StuI
**VT4-F**	5’CGACTC**GGTACC**ATGGCGGACAATGATTCTCTAG3’	KpnI
**VT4-R**	5’CGACTC**GAATTCTCA**CTTGTCATCGTCATCCTTG3’	EcoRI
**hisG I – F**	5’**TGTACA**CCAGTGGTGCATGAACGC3’	BsrGI
**hisG I – R**	5’**ACATGT**CTAGGGATAACAGGGTAATATAGACATGG3’	BsrGI
**hisG II – F**	5’CGACTC**CCCGGG**CCAGTGGTGCATGAACGC3’	XmaI/SmaI
**hisG II – R**	5’CGACTC**CTGCAG**CTAGGGATAACAGGGTAATATAGACATGG3’	PstI
**KanMX-F**	5’CGACTC**TGTACA**CTGAAGCTTCGTACGCTGCA3’	BsrGI
**KanMX-R**	5’CGACTC**CCCGGG**ATCACCTAATAACTTCGTATAGCATACATTATAC3’	SmaI
**GPDADH1-F**	5’CGACTC**CATATG GCGGCCGC**GTCGAAACTAAGTTCTTGGTGTTTTAAAACT3’	NdeI /NotI
**GPDADH1-R**	5’CGACTC**GACGTC AAGCTT**GGCATGCGAAGGAAAATGAGA3’	AatII / HindIII
**KlACT1-F**	5’ATGGATTCTGAGGTCGCTGC3’	
**KlACT1-R**	5’TTAGAAACACTTCAAGTGAACGATGG3’	
**P1**	5’ACACACGTAAACGCGCTCGGT3’	
**P2**	5’ATCATCCTTGTCAGCGAAAGC3’	
**P3**	5’ACCTGAAGATAGAGCTTCTAA3’	
**P4**	5’GGTACCCCTAGGAGATCTAGCTC3’	

Underlined are shown the Flag Tag sequence.

In bold are represented the restriction site.

In grey, the stop codon.

**Table 3:** Estimated copy number (ENC) of AtGME and AtVTC genes by absolute quantification

**Strains**	**C**_**T**_^**a**^			**Copies (Copies.μL**^**-1**^**)**			**ENC**	
	**ACT1**	**GME**	**VTC**	**ACT1**	**GME**	**VTC**	**GME**	**VTC**
**JVC1-51**	20.43 ± 0.37	21.52 ± 0.43	20.19 ± 1.23	2.95E + 04	4.35E + 03	1.61E + 04	1	1
**JVC1-53**	18.92 ± 0.61	19.52 ± 1.69	20.01 ± 0.08	1.34E + 04	4.26E + 04	1.14E + 04	3	1
**JVC1-56**	15.88 ± 2.28	15.43 ± 198	16.72 ± 0.32	1.06E + 05	4.02E + 05	1.70E + 05	4	2
**JVC3-18**	18.68 ± 0.35	20.41 ± 0.17	14.88 ± 0.95	8.21E + 04	1.58E + 04	2.98E + 05	1	4

^a^average ± SD.

**Figure 3:**
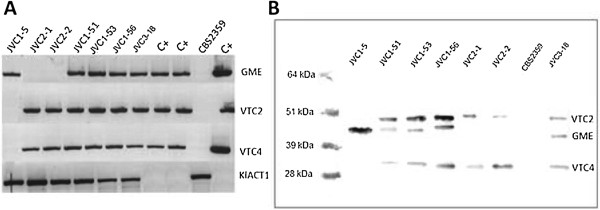
**Expression analyses of L-AA pathway plant genes by recombinant *K. lactis *cells.** (**A**) RT-PCR using cDNA from *K. lactis* cells transformed with three early L-AA pathway plant genes from Arabidopsis *thaliana*. C + − the cDNA from *A. thaliana* leaves and plasmids harboring the corresponding genes were used as controls for RT-PCR analysis. KlACT1 gene from *K. lactis* CBS2359 was used as a control for RNA quality. (**B**) – Western blotting of flag-tagged immunoprecipitated proteins from *K. lactis* recombinant cells using monoclonal anti-flag antibody. *K. lactis* CBS2359 strain was used as negative control. The RNA extraction and total protein extraction were carried out from cells grown in YP medium with 2% (w/v) D-Galactose after 24 hours incubation at 30°C, 200 rpm.

The GME gene is under the control of the inducible LAC4 promoter upon integration by homologous recombination. The strong constitutive *S. cerevisiae* promoters *GPD1* and *ADH1* drive the transcription of the VTC2 and VTC4 respectively. The expression analysis of L-AA pathway plant genes in *K. lactis* recombinant cells was achieved by Reverse Transcriptase-PCR and the flag-tagged proteins from total protein extract were immunoprecipitated before SDS-PAGE, blotted and detected using monoclonal anti-Flag antibodies (Figure 3). All JVC1-5 derived strains, JVC1-51, JVC1-53 and JVC1-56, are expressing the L-galactose pathway genes, GME, VTC2, VTC4. The JVC1-5 only expresses GME and the JVC2-1 and JVC2-2 strains are the control strains for VTC2 and VTC4 expression.

**Figure 4:**
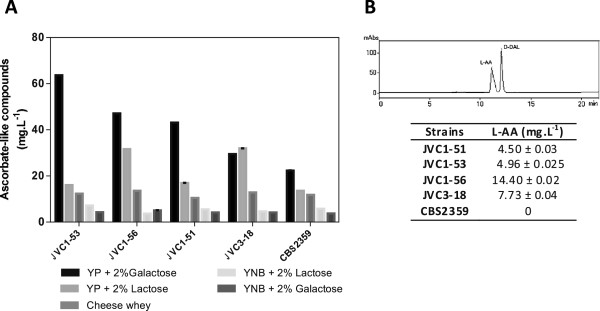
**Intracellular ascorbate-like compounds and L-AA produced by *K. lactis *strains harboring plant genes.** (**A**) Transformed yeast cells were grown on cheese whey, mineral medium (0.67% [w/v] YNB) and on rich (YP) medium (20 g.L^-1^peptone, 10 g.L^-1^ Yeast extract) supplemented with 2% (w/v) D-galactose or lactose, for 48 h (initial OD_600_ 0.05). (**B**) Measurement of L-AA by HPLC Analysis. The retention time for L-AA and D-DAL were 11.175 and 12.003 min respectively when applying a C18 column with 99:1 H_2_O/acetic acid as mobile phase (upper **B**). The strains were grown in YP medium supplemented with 2% (w/v) galactose for 48 hours. CBS2359 parental strain was taken as control.

Simultaneous expression of the proteins GME (43.8 kDa), VTC2 (49 kDa), and VTC4 (30 kDa) in the engineered JVC3-18 and JVC1-5 derived strains should result in the production of L-galactose, when lactose or D-galactose are used as the carbon source in the growth medium. To address whether the plant genes integrated into the *K. lactis* genome would allow the cells to produce L-galactose from GDP- mannose, we analyzed the L-galactose content in the recombinant strains grown in YP medium supplemented with 2% (w/v) D-galactose and in YP medium with 2% (w/v) lactose for 24 hours at 30°C, 200 rpm. Since we could not detect intracellular L/D-Galactose production through HPLC analysis, we believe that the expression, as shown by western blot analysis, of the GME, VTC2, and VTC4 in *K. lactis* cells did not result in any measurable L/D-galactose biosynthesis (data not shown). Perhaps, the L-galactose synthesized was immediately converted into L-AA by the D-DAL enzymes thereby preventing its intracellular accumulation. Hence, the recombinant strains were screened for L-AA production. They were grown for 48 hours in YP or in YNB medium supplemented with 2% (w/v) D-galactose or lactose before the level of L-AA was determined. Figure 4 shows the intracellular L-AA levels produced by the *K. lactis* strains that we engineered in this study. The L-AA assay depends on the ability of ascorbate-like compounds to reduce Fe^3+^. The accumulation of intracellular ascorbate-like compounds in JVC1-5 derived strains or in the JVC3-18 strain was 2 to 3 times higher, but only when cultivated in YP medium and not in minimal medium with D-galactose as carbon source (Figure 4A). When cells were cultivated in both YP and YNB medium with lactose as the sole carbon source, the accumulation was lower, but a two-fold increase was still present in the JVC1-56 and JVC3-18 strains. We also evaluated the ascorbate-like compounds production by culturing recombinant strains in cheese whey, which is the waste product during cheese production, as an alternative lactose source. However, when cheese whey was used as substrate, all recombinant strains showed low intracellular ascorbate-like compounds accumulation. In the untransformed strain, low levels of L-AA/D-DAL could be measured in either minimal or rich medium supplemented with D-galactose. Since this method cannot distinguish between introduced L-AA and the endogenous D-DAL, we identified and measured the L-AA in the recombinant strains by HPLC analysis. Considering their quite similar physical and chemical properties, the L-AA and D-DAL presented a different retention time with about 11.175 and 12.003 min respectively. The strains JVC1-56 and JVC3-18 resulted in higher L-AA production 14.4 and 7.73 mg.L^-1^ respectively when cultivated on YP medium supplemented with 2% (w/v) galactose (Figure 4B).

## Discussion

**Figure 5:**
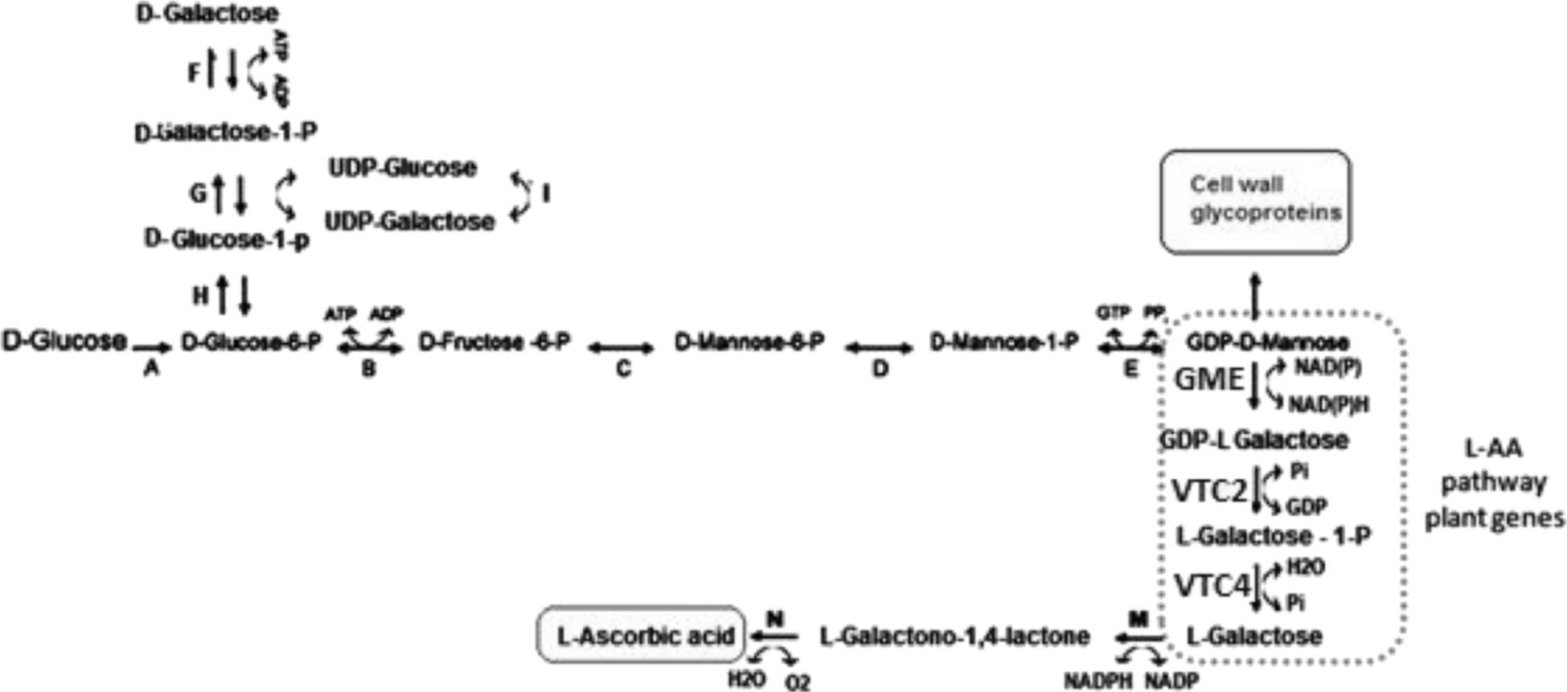
**L-AA pathway engineered in *K. lactis *cells using D-galactose or lactose as substrate.** Lactose is first hydrolyzed by the β-galactosidase enzyme into D-Glucose and D-galactose which are promptly metabolized. A- hexokinase, B - glucose-6-phosphate isomerase, C – mannose-6-phosphate isomerase, D – phosphomannomutase, E – mannose-1-phosphate guanyltransferase, F – Galactokinase, G – galactose-1-phosphate urydyltransferase, H – phosphoglucomutase, I – UDP-galactose-1-epimerase, GME- GDP-mannose 3,5 epimerase, VTC2 – GDP-L-galactose phosphorylase, VTC4- L-Galactose 1-phosphate phosphatase, M – D-arabinose dehydrogenase, N – D-arabinono 1,4 lactone oxydase.

In Figure 5 we present an overview of the L-AA pathway as we have engineered it in *K. lactis*. The insertion of the L-AA pathway plant genes into the *K. lactis* genome creates an alternative route to metabolize GDP-mannose, which is naturally produced in yeasts for cell wall construction [35]. Three other pathways for L-AA production in plants have been described [36]: the L-Gulose pathway [15], the D-Galacturonic acid pathway [11], and the Myoinositol pathway [12], but these seem to be of minor importance. GDP-mannose undergoes epimerization to GDP-L-galactose by GME activity. VTC2 and VTC4 convert GDP-L-galactose in L-galactose that can be used as substrate for L-AA biosynthesis by D-DAL pathway enzymes. The D-DAL pathway is the only known route which contains enzymes able to metabolize non-physiological substrates such as L-galactose [7]. Considering cofactor enzymes requirements, the new GDP-mannose branched pathway apparently does not affect the cell redox balance. The overall GME reaction is redox neutral and uses bound NADP to aid the internal redox reactions needed for the epimerization reactions. Besides the Glutathione/Thioredoxin reductase system, two alternative dehydrogenases in the external mitochondrial membrane (NDE1 and NDE2) are the main source of cytosolic NADPH reoxidation in *K. lactis* cells [37]. NADPH reoxidation is extremely important to maintain the pentose phosphate pathway which has been reported more active in *K. lactis* compared to *S. cerevisiae*[38].

Lactose and galactose metabolism seem to have different effects on this branched pathway. Probably, glucose released from lactose hydrolysis by β-galactosidase activity, may somehow affect the activity of the L-galactose pathway enzymes. When lactose was utilized as carbon source there was a higher ascorbate-like background in *K. lactis* CBS2359 cells (Figure 4A). Although there is no evidence of enzyme activity and metabolites detection, we believe that this background in *K. lactis* CBS2359 is due to D-DAL naturally occurring in this yeast, which is much higher than in *S. cerevisiae*. This idea is also supported by Porro & Sauer, 2003 [39].

Cheese whey represents 85-95% of the milk volume retaining about 85% of milk nutrients such as lactose, soluble proteins, lipids and minerals. It also contains appreciable quantities of lactic and citric acids, non-protein nitrogen compounds (urea and uric acid) and B-group vitamins [40]. The first reaction catalyzed by GMEp for GDP-L-galactose biosynthesis competes for the GDP-mannose with the cell wall glycoproteins pathway enzymes. We suggest that rich medium can provide intermediate metabolites that could be promptly assimilated reducing metabolic flux towards biosynthetic pathways such as cell wall biosynthesis. Likely, the YP medium might enhance the flux of GDP-mannose towards L-galactose formation and its subsequent conversion into L-AA. The HPLC analysis confirmed the L-AA production by the recombinant strains. JVC1-56 resulted in higher L-AA accumulation (14.40 mg.L^-1^) followed by JVC3-18 (7.73 mg.L^-1^). The total L-AA production from all strains obtained in this study was about 30 mg.mL^-1^. Porro et al., 2004 [25] have reported the production of 100 mg.L^-1^ L-AA by recombinant *S. cerevisiae* cells. However, this 3-times higher production was achieved by overexpressing the endogenous D-arabinono-1.4-lactone oxidase gene as well as L-galactose dehydrogenase in the presence of 250 mg.L^-1^ of L-galactose, the main L-AA precursor, in the growth medium. Herein, we report for the first time the production of L-AA in the absence of any L-AA precursors such as L-galactose, L-galactono-1,4-lactone, or L-gulono-1,4-lactone intermediates in the growth medium. The *K. lactis* recombinant strains were able to intracellularly produce L-galactose and convert it into a significant L-AA content without any overexpression of endogenous gene.

The absolute quantification of each cassette into LAC4 promoter locus revealed that the strain JVC1-56 harbors four copies of the GME gene. GME encodes the GDP-mannose 3,5- epimerase that catalyzes the conversion of GDP-mannose into GDP-L-Galactose, the first reaction which competes for GDP-mannose with cell wall glycoprotein biosynthesis. Thus, the high expression level of GMEp ensures the metabolic flow throughout the L-AA biosynthetic engineered pathway. Cheese whey represents an environmental problem due to its high volumes produced. In addition, the high organic matter content, mainly lactose, exhibits a biochemical oxygen demand (BOD) of 30–50 g.L^-1^ and a chemical oxygen demand (COD) of 60 – 80 g.L^-1^[40,41]. As cheese whey permeate is not as rich as supposed once it loses most of the whey protein at ultrafiltration process, the ascorbate-like compounds accumulation was lower when the cells were grown in this medium. Nevertheless, the recombinant *K. lactis* strains can still convert lactose from whey to valuable compounds such as L-AA based on their fermentation capacity. Moreover, since L-AA acid-producing yeast strains have an improved stress resistance and robustness [26], these strains may also be used as host for producing heterologous proteins with industrial interest in biotechnological processes, in case it is shown that our recombinant *K. lactis* strain is also more tolerant to these conditions.

The downstream L-galactose metabolism could be the bottleneck for L-AA biosynthesis throughout this engineered pathway since D-DAL enzymes regulation in yeast has not extensively been elucidated. The D-DAL production is observed when yeasts are grown on some sources of D-aldoses such as D-glucose, D-galactose, D-mannose or D-arabinose [42]. The kinetic parameters of D-arabinose dehydrogenase (Ara2) and D-arabinono-1, 4 lactone oxydase (Alo1) have been determined *in vitro* and the results have demonstrated low substrate specificity [43]. The Alo1 enzyme has a putative domain for the covalent FAD molecule similar to the domain found in oxygen-dependent oxidoreductases. Spickett et al. (2000) [44] found that the production of L-AA analogues is strongly influenced by the aeration of the culture. Probably the key regulatory enzyme, Alo1p, may be dependent on the dissolved oxygen levels. Besides, this enzyme seems to play a role in oxidative stress response. When the *S. cerevisiae alo1*Δ strain was grown in the presence of H_2_O_2_, cells were more sensitive while the overexpression leads to resistance. However no changes in the transcription levels of the *ALO*1 gene were observed under the same conditions. Thus, transcriptional and post translation regulation of the genes from D-DAL pathway in yeast must be considered in this process. Thus, a better understanding about the regulation and functionality of the D-DAL biosynthetic genes in *K. lactis*, might be the main target in order to improve L-AA biosynthesis.

## Conclusions

This work is the first attempt of engineering *K. lactis* cells for L-ascorbic acid biosynthesis by fermentation taking advantage of its natural ability to grow on lactose and without any exogenously addition of its precursors in the growth medium. By the insertion of the L-galactose pathway genes from *A. thaliana*, we engineered *K. lactis* strains capable of converting lactose and D-galactose into L-galactose, a rare sugar which is one of the main precursors for L-AA production.

## Methods

### Strains and growth conditions

*Escherichia coli* TOP10 cells [F- mcrA Δ(mrr-hsdRMS-mcrBC) φ80lacZΔM15 ΔlacX74 nupG recA1 araD139 Δ(ara-leu)7697 galE15 galK16 rpsL(Str^R^) endA1 λ^-^] were used to amplify the plasmids. *E. coli* cells were grown on Luria Bertani (LB) medium (10 g.L^-1^ tryptone, 5 g.L^-1^ yeast extract, 10 g.L^-1^ NaCl, pH 7.5) with or without 100 μg.mL^-1^ ampicillin at 37°C. *E. coli* TOP10 cells harboring the vector pGEM T easy were grown on solid LB medium supplemented with 1 mM isopropyl β-D-thiogalactopyranoside (IPTG) and 40 μg.mL^-1^ 5-bromo-4-chloro-3-indolyl- beta-D-galactopyranoside (X-Gal). *Kluyveromyces lactis* CBS2359 strain was used as host for protein expression on this work. YPD medium (20 g.L^-1^peptone, 10 g.L^-1^ Yeast extract, 20 g.L^-1^Dextrose) or YPGal (20 g.L^-1^peptone, 10 g.L^-1^ Yeast extract, 20 g.L^-1^ Galactose) were routinely used for obtaining biomass of the recombinant and parental yeast strains at 30°C. For solid medium 20 g.L^-1^ agar was added. YCB (Yeast Carbon Base - Sigma) medium supplemented with 5 mM acetamide and YPD containing 200 μg.mL^-1^ geneticin were used to select *K. lactis* cells transformed with the vectors constructed on this work. Cheese whey, YNB (Yeast Nitrogen Base - Sigma) or YP medium supplemented with 20 g.L^-1^ galactose or lactose was used to grow the cells for ascorbate-like compounds and L-AA measurements.

### L-ascorbic acid pathway genes amplification

L-AA pathway genes from *Arabidopsis thaliana*, GDP-D-Mannose 3',5'-Epimerase [GME (E.C. 5.1.3.18)], GDP-L-Galactose Phosphorylase [VTC2(E.C.2.7.7.220], L-Galactose-1-Phosphate Phosphatase [VTC4 ( E.C. 3.1.3.23) were amplified using *A. thaliana* cDNA, kindly provided by Dr. Filip Rolland (K.U. Leuven, Belgium), as a template. Phusion High Fidelity DNA polymerase was used for PCR amplification and primers are listed in Table 2. Amplification cycles comprised 5 minutes 95°C, 1 minute 95°C, 30 seconds Tm^x^, 90 seconds 72°C, 5 minutes 72°C. Tm^x^ was 58°C for GME, 66°C for VTC2 and 60°C for VTC4 amplification. L-AA pathway genes were tagged with the Flag Tag (Asp-Tyr-Lys-Asp-Asp-Asp-Asp-Lys) by adding the corresponding DNA sequence in each primer (Table 2).

### Construction of expression cassettes

Maps of the plasmids used in this study are shown in Figure 1. pKLAC1 plasmid [30] was used as starting point. pMB7-A [45] was used as template for hisG fragments amplification, 1 minute 94°C, 1 minute 63°C, 1 minute 68°C (34 cycles), with the primers hisGI-F and hisGI-R, hisGII-F and hisGII-R. HisG fragments were subcloned into pGEM T easy Vector and further transferred to pKLAC1 generating the plasmid pKLhisG2. The repeat hisG sequences flank the *amdS* (acetamidase) marker for its removal by homologous recombination in the counterselection procedure. Bidirectional promoter in the pBEVY-L vector [46], *ScGPD1* and *ScADH1*, and the *ADH2* terminator sequence were amplified using the primers GPDADH1-F and GPDADH1-R in the following amplification cycles: 20 sec 98°C, 20 sec 63°C, 45 sec 72°C (34 cycles). The resulting 1405 bp fragment was subcloned into the pGEM vector linearized by AatII and NdeI, generating the vector pGDPADH1. The AtVTC4 gene was inserted into pGPDADH1 linearized by EcoRI and KpnI. Finally, the AtVTC4 expression cassette, under the control of the ADH1 promoter, was cut out from the pGPDADH1 vector and cloned into pKLhisG2, linearized with HindIII and NotI. Afterwards, the AtVTC2 gene was released from the pGEM Vector with NotI and StuI digestion and transferred to pKLhisG2, linearized with the same restriction sites resulting in the vector pKlVTc. pKLAC1 was digested with HindIII and XhoI, followed by treatment with Klenow enzyme and also with T4 DNA ligase to destroy the signal secretion sequence of the alpha mating factor. The AtGME gene was released from the pGEM vector by cutting with XhoI and StuI and inserted into the SalI and StuI sites from pKLAC1 α-mating factor free vector generating the vector pKLJC/GME. The LoxP-KanMX-LoxP cassette was amplified by PCR using pYX012 (Novagen) as a template and the primers KanMX-F and KanMX-R in the following amplification cycles: 3 minutes 98°C, 20 sec 98°C, 20 sec 63°C, 45 sec 72°C (34 cycles). The cassette was further inserted into BsrGI and XmaI site from pKLJC/GME vector. All ligation reactions were performed with Rapid DNA Ligation Kit from Roche®.

### Yeast transformation

*Kluyveromyces lactis* transformation was carried out according to Kooistra et al*.* 2004 [47], with some modifications. Fresh CBS2359 cells were plated on YPD agar medium and incubated overnight at 30°C. An isolated colony was grown in 2 mL YPD culture at 30°C, 200 rpm overnight. 50 mL YPD were inoculated with these 2 mL pre-cultured cells to start O.D_600_ 0.0025 per mL (0.1 OD). When O.D_600_ reached approximately 1, the cells were harvested at 3000 rpm for 5 minutes at 4°C and washed with 25 mL sterile ice-cold electroporation buffer EB (10 mM Tris–HCl, pH 7.5, 270 mM sucrose and 1 mM MgCl_2_). 25 mL YPD medium containing 25 mM DTT and 20 mM HEPES pH 8.0 were added and further incubated at 30°C for 30 minutes without shaking. Cells were collected at 3000 rpm for 5 minutes at 4°C and washed with 10 mL sterile ice-cold EB buffer. Cells were resuspended in 0.2 mL ice-cold EB and added to 60 μL aliquots of competent cells. To each aliquot 50 μg Salmon Sperm DNA (SS-DNA) plus 2 μg transforming DNA was added and kept on ice for 15 minutes. The mixture was transferred to a chilled electroporation cuvette (2 mm) and electroporated at 1 KV, 25 μF, and 400 Ohm. Immediately, 1 mL YPD was added and the mixture was incubated at 30°C for 3 hours, 200 rpm. The cells were harvested at 3000 rpm for 5 minutes at 4°C and washed with sterile water. Cells were plated on selective agar plates and kept at 30°C for 2 days.

### Total DNA extraction and yeast transformants screening

Cells were grown in 2 mL YPD at 30°C to saturation. Biomass was collected by centrifugation, resuspended in 0.2 mL lysis buffer (2% Triton X-100, 1% SDS, 100 mM NaCl, 10 mM Tris pH8, 1 mM EDTA) and transferred to a 2 mL screwcap tube. Afterwards, 0.2 mL PCI [phenol pH 6.7- chloroform-isoamylalcohol (25:24:1)] and 0.3 g glass beads were added. The cells were broken using the fastprep machine, speed 6 for 20 sec followed by centrifugation at 14,000 rpm for 10 minutes. The supernatant was transferred to a new tube; 0.5 mL ethanol was added and kept at −20°C for at least 20 minutes. The total DNA was pelleted by centrifugation at 14,000 rpm for 10 minutes, washed with 70% ethanol and dried at room temperature. The DNA samples were dissolved in 30 μL nuclease-free H_2_0 and kept at −20°C. The correct cassette integration into the LAC4 locus was confirmed by colony PCR or by using their total DNA as template. For colony PCR, isolated colonies obtained on selective media were transferred to fresh selective agar media for the isolation of single colonies. Single colonies were picked up with a sterile toothpick and dissolved in 100 μL 0.01 M NaOH and kept at room temperature for 45 minutes. A 1.5 μL aliquot of this sample or 1 μL from total purified DNA was used as a template for a 50 μL PCR reaction. The specific primers used to detect the single or multiple cassette insertions into the LAC4 promoter locus are indicated in Table 1. The amplification cycles comprised 5 minutes 98°C, 45 seconds 98°C, 30 seconds 58°C, 1 minute 72°C (35 cycles), and 5 minutes 72°C.

### Integrated cassette absolute quantification

The ACT1 gene, which is a single-copy gene in *K. lactis* chromosomal DNA, was amplified from the *K. lactis* CBS2359 strain and used as reference to normalize the data. The PCR product was purified using the GenElute™ PCR Clean-Up Kit (Sigma-Aldrich™) and cloned into pGEM T Easy vector (Promega, Madison, WI, USA). The vectors pKlJC/GME (9215 bp) and pKlVTc (13827 bp) harboring the AtGME and AtVTC2/AtVTC4 genes respectively, plus pGEM/Act1 were used to construct the standard curves for DNA absolute quantification of the yeast transformants. Genomic DNA from each strain and the vectors constructed in this study were quantified using NanoDrop 2000 (Thermo Fisher Scientific Inc, USA) and diluted to 10 ng.μl^-1^. The real-time PCR analysis was performed in 96-well optical plates in technical triplicates with primers designed using Primer3 software [48]. 2 μl of the diluted DNA or plasmid DNA dilutions, 0.2 μM of forward and reverse primer, and Platinum® SYBR® Green qPCR Super Mix-UDG (Invitrogen) in a 1 X final concentration, were added for a 25 μl final volume reaction. The CFX96™ Real-Time PCR Detection System (BioRad) was used as follows; 2 min at 50°C, then 2 min at 95°C followed by 40 cycles of 15 s at 95°C and 30 s at 60°C. The conversion of mass concentration of the vector to copy concentration was done following the equation [49]:

$$\mathrm{DNA}\;\left(\mathrm{copy}\right)\frac{=6.02\;\times \;10^{23}\left({\mathrm{copies}\;\mathrm{mol}}^{-1}\right)\;\times \;\mathrm{DNA}\;\mathrm{amount}}{\mathrm{DNA}\;\mathrm{length}\;\left(\mathrm{bp}\right)\;\times \;660\;\left({g\;\mathrm{mol}}^{-1}{\mathrm{bp}}^{-1}\right)}$$

A tenfold serial dilution was used for all plasmids to construct the standard curves, with pGEM/Act1 ranging from 6 × 10^2^ to 6 × 10^8^ copies.μl^-1^, pKlJC/GME ranging from 4 × 10^2^ to 4 × 10^8^ copies.μl^-1^, and pKlVTc ranging from 3 × 10^2^ to 3 × 10^8^ copies.μl^-1^. With these calculations, the precise number of molecules added to subsequent real-time PCR runs was calculated, providing a standard for copy number quantification of AtGME and AtVTC2/VTC4 genes. The C_T_ values were plotted against the log of the number of molecules and each standard curve was generated by a linear regression. By relating the C_T_ value to a standard curve it was possible to determine the exact copy concentration of the target gene. After determining the standard curve, the standard plasmid dilutions were performed simultaneously in a run with the total DNA samples from the yeasts transformants. The AtGME and AtVTC2/VTC4 copy number was calculated by dividing the copy concentration of these genes by that of *ACT*1 gene. The experiments were performed in biological triplicate using three preparations of total DNA from independent biological samples.

### Total RNA extraction from yeast and RT-PCR

The cells were grown overnight in 5 mL YPGal medium at 30°C, 250 rpm. The cells were pelleted by centrifugation and the supernatant was discarded. The total RNA from recombinant *K. lactis* yeast cells was extracted using the Trizol® method (Invitrogen). The cDNA synthesis from the total RNA extracted was achieved using the Reverse Transcription System from Promega®. A 2 μL cDNA aliquot from each sample was used in a 50 μL PCR reaction in order to qualitatively detect mRNA expression of the L-AA pathway plant genes inserted into *K. lactis* genome. The RT-PCR was performed using the same primers and amplification cycles used for plant genes amplification.

### Protein extraction, immunoprecipitation and western blotting

The recombinant cells were precultured overnight in 3 mL YPGal, 20 rpm at 30°C and used to inoculate 50 mL YPGal. When the culture reached the OD_600_ of 5, the cells were pelleted by centrifugation at 3,000 rpm, 4°C for 5 minutes and washed with ice-cold Phosphate buffered saline (PBS, 140 mM NaCl, 2.7 mM KCl, 10 mM Na_2_HPO_4_, 1.8 mM KH_2_PO_4_ at pH 7.3). Protein extraction was carried out with glass beads in lysis buffer containing 1× PBS, 0.001% Triton X-100, 8.7% glycerol, 25 mM MgCl_2_, 10 mM EDTA (pH 7), 10 mM dithiotreitol, 100 mM NaF, 4 mM Na_3_VO_4_, 1 mM β-glycerophosphate and one tablet of Complete Protease Inhibitor Cocktail (Roche). Total protein content was measured according to Bradford, 1975 using bovine serum albumin (BSA) as standard. An aliquot, comprising 400 to 500 μg total protein extract, was used for flag tagged protein immunoprecipitation with monoclonal anti-FLAG antibodies (M2, Sigma-Aldrich) by incubation with Protein G agarose (Roche) for 3 hours at 4°C. SDS sample buffer (5X: 250 mM Tris–HCl, 10% SDS, 0.5% bromophenol blue, 1.4 M β-mercapto-ethanol) was added after three wash steps and stored at −20°C.

Proteins were separated by SDS-polyacrylamide gel electrophoresis on the NUPAGE Novex Bis-Tris mini Gel system (Invitrogen®). Separated proteins were transferred to nitrocellulose membrane (HybondC extra, Amersham) and detected by incubation with monoclonal anti-Flag antibodies and horseradish peroxidase-conjugated anti-mouse IgG secondary antibodies (Amersham) and detected using the Supersignal West Pico Luminol solution (Thermo Scientific). Immunoblots' chemiluminescence was imaged using Fujifilm LAS-4000 mini, and the accompanying software Image Reader LAS-4000 (Life Science Fuji Photofilm Co., Ltd).

### Measurement of intracellular L-galactose formation

Recombinant cells precultured in 3 ml YPGal were used to inoculate 50 mL YPGal, 30°C, 200 rpm for 24 hours. The cells were harvested by filtration on nitrocellulose filters 0.45 μm, transferred to 8 mL methanol/chloroform (5 mL MeOH/3 mL Chloroform) and kept at −20°C overnight. Aliquots from the supernatant were taken, transferred to 2 mL tubes and cleared by centrifugation at 12,000 rpm at 4°C for 10 minutes. Fractions of the supernatant were dried by speedvac and resuspended in 1 mL milliQ H_2_O. Charged compounds were removed from the sample using Dowex ion-exchange resins (1:1 v/v) 50WX8-200 (Sigma-Aldrich) and 1×8 200 (Acros Organics) The samples were used immediately for HPLC analysis (CarboPac PA1 anion-exchange column, 10 μm, 4 × 250 mm, DIONEX, eluent: 100 mM and 16 mM NaOH, flow rate: 1 mL.min^-1^, detection: pulse amperometry ED40 gold electrode) using pure D-galactose (Sigma-Aldrich, G0750) and L-galactose (Sigma, G7134) as standards.

### Determination of ascorbate-like compounds and L-Ascorbic acid

For intracellular L-ascorbic acid determination, yeast cells were pregrown in 3 mL YP or YNB medium supplemented with 2% (w/v) galactose or lactose. These cells were used to inoculate 50 mL of either medium at an initial optical density of 0.1. The cells were grown for 24 hours, harvested by centrifugation at 5000 rpm for 5 minutes at 4°C and washed once with ice cold distilled H_2_O. The cell pellet was resuspended in about twice the volume with ice cold 10% (w/v) trichloroacetic acid, vortexed vigorously for 2 min and kept on ice for 20 minutes. The supernatant was cleared from cell debris by centrifugation. Ascorbate-like compounds were determined spectrophotometrically according the method adapted from Sullivan et Clarke (1955) [50]: 135 μL of sample was mixed with 40 μL 85% (v/v) H_3_PO_4_, 675 μL 0,5% (w/v) α’α’ dipyridyl and 135 μL 1% (w/v) FeCl_3_. After incubation at room temperature for 10 minutes the absorbance at 525 nm was measured. The identity and L-AA measurements were achieved by high performance liquid chromatography with Luna 5u C18 column (250 × 4.6 mm, Phenomenex) with 99:1 H2O/acetic acid as eluent, a flow rate of 0.5 mL.min^-1^, and UV detection set at 254 nm and the L-AA content was calculated using the L-AA standard curve. The L-AA (cat. nº A5960) and D-DAL (cat. nº 58320) standard curve was made using reagents from sigma Aldrich.

### Statistical analysis

The ascorbate-like compounds and L-AA measurement experiments were carried out at least three times. Herein, we reported mean values as well as for L-AA standard curve. Student’s *t*-test was performed with p < 0.05.

## Abbreviations

L-AA: L-ascorbic acid; AtGME: *Arabidopsis thaliana* GDP-mannose-3,5-epimerase; AtVTC2: *Arabidopsis thaliana* GDP-L-galactose phosphorylase; AtVTC4: *Arabidopsis thaliana* L-galactose-1-phosphate phosphatase; D-DAL: Dehydro-D-arabinono-1,4-lactone; ScGPD1: *Saccharomyces cerevisiae* Glycerol-3-phosphate dehydrogenase; ScADH1: *Saccharomyces cerevisiae* alcohol dehydrogenase; LDGH: L-galactose dehydrogenase; ALO1: D-arabinose-1,4-lactone oxidase; AGD: L-galactona-1,4-lactone dehydrogenase; FGT: L-fucose guanylyltransferase; LAC4: β-galactosidase; BOD: Biochemical oxygen demand; COD: Chemical oxygen demand; EB: Electroporation buffer; SS-DNA: Salmon sperm DNA; RT-PCR: Reverse transcriptase PCR.

## Competing interests

The authors declare that they have no competing interests.

## Authors’ contributions

FMLP conceived the idea. JCCR and PVD have designed all experimental strategy. JCCR performed most of the experiments. JCCR, PVD and FMLP have equally contributed to the interpretation of data and to the preparation of the current version of this submission. NA contributed with the vector construction, LTC performed the absolute quantification measurements, MCTA performed the L-AA HPLC analysis. All authors read and approved the final manuscript.
